# EbsA is essential for both motility and biofilm formation in the filamentous cyanobacterium Nostoc punctiforme

**DOI:** 10.1099/mic.0.001498

**Published:** 2024-09-17

**Authors:** Aya S. Hassan, Ethan S. Heflen, Khoa D. Nguyen, Gabriel A. Parrett, Douglas D. Risser

**Affiliations:** 1 Department of Biology, University of Colorado Colorado Springs, Colorado Springs, CO 80918, USA

**Keywords:** biofilm, cyanobacteria, extracellular polymeric substance (EPS), motility, type IV pilli

## Abstract

Many cyanobacteria, both unicellular and filamentous, exhibit surface motility driven by type IV pili (T4P). While the component parts of the T4P machinery described in other prokaryotes are largely conserved in cyanobacteria, there are also several T4P proteins that appear to be unique to this phylum. One recently discovered component is EbsA, which has been characterized in two unicellular cyanobacteria. EbsA was found to form a complex with other T4P proteins and is essential for motility. Additionally, deletion of *ebsA* in one of these strains promoted the formation of biofilms. To expand the understanding of *ebsA* in cyanobacteria, its role in motility and biofilm formation were investigated in the model filamentous cyanobacterium *Nostoc punctiforme*. Expression of *ebsA* was strictly limited to hormogonia, the motile filaments of *N. punctiforme*. Deletion of *ebsA* did not affect hormogonium development but resulted in the loss of motility and the failure to accumulate surface pili or produce hormogonium polysaccharide (HPS), consistent with pervious observations in unicellular cyanobacteria. Protein-protein interaction studies indicated that EbsA directly interacts with PilB, and the localization of EbsA-GFP resembled that previously shown for both PilB and Hfq. Collectively, these results support the hypothesis that EbsA forms a complex along with PilB and Hfq that is essential for T4P extension. In contrast, rather than enhancing biofilm formation, deletion of both *ebsA* and *pilB* abolish biofilm formation in *N. punctiforme*, implying that distinct modalities for the relationship between motility, T4P function and biofilm formation may exist in different cyanobacteria.

## Data Summary

Supplementary material is available with the onine version of this article, available through Figshare at https://doi.org/10.6084/m9.figshare.26799265 https://doi.org/10.6084/m9.figshare.26799265 [1][1].https://figshare.com/s/8735d05ce99b3b7c1a37.

## Introduction

Many cyanobacteria, both unicellular and filamentous, exhibit surface motility driven by type IV pili (T4P) (for review see [2,3]). This motility can be modulated by systems that sense light quality and quantity so that the organism can position itself in the optimal light environment to maximize photosynthesis while minimizing photodamage, a process generally referred to as phototaxis [2,3]. This motility also enhances undirected dispersal to new environments. In filamentous cyanobacteria surface motility is also critical for several other biological functions, including the formation of colonial aggregates [4,7] and the establishment of nitrogen-fixing symbioses with plants [5,8].

Type IV pilus systems are broadly conserved among diverse members of the eubacterial domain, particularly diderms [9]. The pilus is assembled from monomers of a major pilin (PilA), and to a lesser extent structurally similar minor pilins, and transverses the outer membrane of Gram-negative bacteria through a gated channel formed by the secretin protein PilQ. The motor complex that powers cycles of pilus extension and retraction to facilitate motility is located in the inner membrane and cytoplasm. Extension of the pilus occurs when the motor ATPase PilB associates with the motor complex and drives rotation of the inner membrane protein PilC, resulting in the incorporation of new pilin monomers at the base of the pilus. Conversely, when the motor ATPase PilT associates with the motor complex it drives rotation of PilC in the opposite direction, resulting in the removal of pilin monomers from the pilus base and thus retraction of the pilus. An alignment complex composed of the periplasmic proteins PilN, PilO, and PilP, and the cytoplasmic protein PilM coordinates the positioning of the motor complex with the outer membrane PilQ channel. The T4P systems in cyanobacteria contain homologs to most T4P proteins characterized in other bacteria, except for the alignment complex protein PilP [2], and typically encode a pair of PilT homologs designated PilT1 and PilT2 [2]. PilT1 appears to be the primary pilus retraction ATPase, as mutations that inactivate *pilT1* abolish motility, while those that inactivate *pilT2* either produce an inverted phototaxis response or reduce motility [10,11].

However, while current data clearly indicate that cyanobacterial T4P systems contain similar components and function in a similar manner as in other bacteria, it has become apparent that there are also several proteins unique to cyanobacteria that participate in the T4P system and are required for motility. One of these is the cyanobacterial homolog of *E. coli* Hfq. While Hfq in *E. coli* functions as an RNA chaperone [12], the available evidence does not support such a role in cyanobacteria [13]. In contrast, the cyanobacterial Hfq homolog interacts directly with PilB and is required for pilus extension [14,16]. The role of Hfq in T4P function has been reported in both unicellular and filamentous cyanobacteria indicating that this is likely universal in motile cyanobacteria.

A second component novel to cyanobacteria is HmpF. Homologs of HmpF are confined to the cyanobacterial phylum and lack any characterized domains but are frequently encoded adjacent to other genes of the Hmp chemotaxis-like system [17]. HmpF is essential for motility, and like Hfq and PilB, HmpF is also required for pilus extension [17,19]. HmpF has been shown to interact with Hfq and PilT proteins [16]. However, unlike Hfq and PilB, which display static bi-polar localization in the filamentous cyanobacterium *N. punctiforme* [11,16], HmpF accumulates only at the leading poles in motile filaments, and filament reversals are preceded by the dissociation of HmpF from the prior leading poles followed by accumulation at the new leading pole [16]. The dynamic localization of HmpF is dependent on other components of the Hmp chemotaxis system [16]. Collectively, this data supports a model where the Hmp chemotaxis system modulates the association of HmpF with the T4P motors, activating the motors at one side of the cell to facilitate directional motility. Notably, in unicellular cyanobacteria dynamic localization of PilB associated with the direction of motility has been observed [20], implying that regulation of directional motility may be distinct in unicellular versus filamentous cyanobacteria.

A third novel component is the DnaK1/DnaJ3 chaperone system. In *N. punctiforme* this system co-localizes with the T4P motors, with DnaJ3-GFP showing static, bipolar localization and DnaK1-GFP exhibiting dynamic unipolar localization similar to that observed for HmpF [21]. Protein-protein interaction studies demonstrated that DnaK1 interacts with both DnaJ3, PilB, and Hfq [21]. However, while mutation of *dnaK1* and *dnaJ3* dramatically reduce motility, they do not completely disrupt it, and the evidence indicates that the T4P systems remain functional. Instead, these mutants are deficient in production of the motility-associated hormogonium polysaccharide (HPS), indicating that this chaperone system may serve as a regulatory link between T4P activity and HPS secretion [21]. The *dnaK1* and *dnaJ3* ortholog in *Synechocystis* sp. PCC 6803 (hereafter *Synechocystis*) have been shown to affect motility [18,22], and protein-protein interaction studies indicate that the DnaK1 ortholog interacts with the T4P motors as well [21]. These findings suggest that the chaperone system functions to coordinate T4P activity with polysaccharide production.

The final protein unique to cyanobacteria that was found to be a part of the T4P system is EbsA. This protein was first identified in a genetic screen for mutant strains of the unicellular cyanobacterium *Synechococcus elongatus* sp. PCC 7942 (hereafter *S. elongatus*) with enhanced biofilm formation [23]. This phenotype is similar to that observed for a *pilB* mutant of *S. elongatus* [23]. Coimmunoprecipitation subsequently demonstrated that both PilB and Hfq were enriched in EbsA pull-down experiments, and that this trio of proteins was enriched when PilB or Hfq were used as bait as well, indicating that EbsA forms a complex along with PilB and Hfq [23]. While the strain of *S. elongatus* used in these experiments has lost motility due to laboratory domestication [24], subsequent studies in the motile unicellular cyanobacterium *Synechocystis* demonstrated that like *pilB* and *hfq*, *ebsA* is essential for motility [23]. However, mutation of *ebsA* in *Synechocystis* did not influence biofilm formation [23].

Phylogenetic analysis indicates that like *pilB* and *hfq*, orthologs of *ebsA* are widespread in cyanobacteria [23], but the role of *ebsA* has not been investigated in any filamentous cyanobacterium. Moreover, while T4P activity and biofilm formation appear to display an inverse correlation in *S. elongatus*, the opposite correlation has been observed in several filamentous cyanobacteria, where motility and thus T4P activity is correlated with biofilm formation [25] and aggregation [4,7]. This suggests an alternate modality for the relationship between motility, T4P activity, and biofilm formation in at least some filamentous cyanobacteria. To explore the role of *ebsA* in a filamentous cyanobacterium, we used a combination of genetic, immunological, cytological, and protein-protein interaction approaches to investigate the function of *ebsA* in the model filamentous cyanobacterium *N. punctiforme*. The results indicate that *ebsA* is essential for both motility and biofilm formation in this organism.

## Methods

### Strains and culture conditions

For a detailed description of the strains used in this study refer to Table S1, available in the online version of this article. * N. punctiforme* ATCC 29133 and its derivatives were cultured in Allan and Arnon medium diluted four-fold (AA/4), without supplementation of fixed nitrogen, as previously described [26], with the exception that 4 and 10 mM sucralose was added to liquid and solid medium, respectively, to inhibit hormogonium formation [27]. For hormogonium induction for phenotypic analysis, the equivalent of 30 µg chlorophyll *a* (Chl *a*) of cell material from cultures at a Chl *a* concentration of 10–20 μg ml^−1^ was harvested at 2 000 ***g*** for 3 min, washed two times with AA/4 and resuspended in 2 ml of fresh AA/4 without sucralose. For selective growth, the medium was supplemented with 50 μg ml^−1^ neomycin. *Escherichia coli* cultures were grown in lysogeny broth (LB) for liquid cultures or LB supplemented with 1.5% (w/v) agar for plates. Selective growth medium was supplemented with 50 μg ml^−1^ kanamycin, 50 μg ml^−1^ ampicillin, and 15 μg ml^−1^ chloramphenicol.

### Plasmid and strain construction

For a detailed description of the plasmids, strains, and oligonucleotides used in this study refer to Table S1. All constructs were sequenced to insure fidelity.

To construct plasmid pDDR557 for in-frame deletion of *ebsA*, approximately 900 bp of flanking DNA on either side of the gene and several codons at the beginning and end of the gene were amplified via overlap extension PCR using primers NpF4125-5′-F, NpF4125-5′-R, NpF4125-3′-F and NpF4125-3′-R, and cloned into pRL278 [28] as a BamHI-SacI fragment using restriction sites introduced on the primers.

To construct plasmid pGAP100 for replacement of the chromosomal allele of *ebsA* with a C-terminal *gfpuv*-tagged variant, approximately 900 bp of DNA downstream of the stop codon were amplified via PCR using primers NpF4125-gfp-3′-F and NpF4125-3′-R and cloned into pSCR569 [29], as an SpeI-SacI fragment using restriction sites introduced on the primers. The coding region of *ebsA* and approximately 900 bp of DNA upstream of the start codon were then amplified via PCR using primers NpF4125-5′-F and NpF4125-gfp-5′-R and cloned into this plasmid as a BamHI-SmaI fragment using restriction sites introduced on the primers.

To construct plasmid pASH100, a mobilizable shuttle vectors containing *ebsA* expressed from the *petE* promoter, the coding-region of *ebsA* was amplified via PCR using primers NpF4125-BamHI-F and NpF4125-SacI-R and subsequently cloned into pDDR155 [5] as a BamHI-SacI fragment, replacing the *hmpA-gfp* coding region, using restriction sites introduced on the primers.

To construct plasmids encoding proteins of interest fused to either the T18 or T25 fragment of *Bordetella pertussis* adenylate cyclase for BACTH analysis [30,31], the coding region of each gene was amplified via PCR (using primers NpF4125-TH-BamHI-F and NpF4125-TH-KpnI-R for *ebsA* and NpF0122-TH-BamHI-F and NpF0122-TH-KpnI-R for *dnaK1*) and cloned into either pUT18/pUT18c or pKT25/pKNT25 as BamHI-KpnI fragments using restriction sites introduced on the primers.

Gene deletion was performed as previously described [32] with *N. punctiforme* cultures supplemented with 4 mM sucralose to inhibit hormogonium development and enhance conjugation efficiency [33, 27]. To construct UCCS108 and UCCS115, plasmids pDDR557 and pGAP110, respectively, were introduced into wild-type *N. punctiforme* ATCC29133.

### Motility assays

Plate and time lapse motility assays were performed as previously described [11]. Briefly, for plate motility assays, colonies were transferred from AA/4 solid medium (1% noble agar) containing 5% sucrose, to suppress hormogonium development, to the surface of AA/4 solid medium (0.5% noble agar) without sucrose. Plates were incubated for 5 days under light. For time lapse motility assays, following standard hormogonium induction from liquid cultures, 2 µl of culture was spotted onto the surface of AA/4 solid medium (0.5% noble agar), overlayed with a cover slip, and imaged at 15 s intervals. Both plate and time lapse motility assays were imaged with a Leica SD9 dissecting microscope equipped with a Leica Flexcam C3 camera controlled by Leica LAS X software. All assays were repeated in triplicate, with representative images and videos depicted.

### Immunoblot and lectin blot analysis

Preparation of *N. punctiforme* cell material, protein extraction and detection of PilA, RbcL, HmpD, and GFPuv by immunoblot analysis was performed as previously described [17]. Briefly, total cellular protein was extracted from cell material equivalent to 30 µg Chl*a* following standard protocols [17], lysate containing extracted proteins was separated on a 4–12% SDS-PAGE gel, and then transferred to a nitrocellulose membrane. Polyclonal antibodies raised against PilA [5], HmpD [33], and RbcL [34] were used at a 1 : 10 000 dilution, followed by a 1 : 20 000 dilution of an HRP-conjugated anti-rabbit secondary antibody (Chemicon). Lectin blot analysis to detect soluble HPS was performed as previously described [11]. Briefly, 100 µl of cell-free culture medium was vacuum transferred to a nitrocellulose membrane and HPS was detected using biotinylated Ulex Europaeus Agglutinin I (UEA) (Vector Laboratories) following standard protocols [11].

### Immunofluorescence and fluorescent lectin staining

Detection of surface PilA and cell-associated HPS by immunofluorescence and fluorescent lectin staining was performed as previously described [11, 17]. Briefly, cells were fixed in 4% paraformaldehyde, followed by methanol and acetone fixation, and subsequently polyclonal α-PilA antibodies [5] and UEA-fluorescein (Vector Laboratories) were used to detect PilA and HPS respectively following standard protocols [5,17].

### Biofilm assays

To perform biofilm assays, 30 µg Chl *a* of cell material from a dense culture was transferred to 50 ml of fresh AA/4 medium in a 125 ml Erlenmeyer flask and incubated under light with shaking at 120 r.p.m. for 3 weeks. Weekly, the cultures were vigorously pipetted to disperse the colonial aggregates that form when strains produce hormogonia. For consistency, the Δ*ebsA* and Δ*pilB* strains were also vigorously pipetted although they do not form colonial aggregates like the wild-type strain. After 3 weeks, the cultures were removed from the flasks, the flasks were washed twice with 10 ml of sterile water and subsequently images were taken of the flasks. After imaging, the flasks were washed with 10 ml of methanol to extract the Chl *a* from cell material adhering to the flask. Quantification of Chl *a* was subsequently performed by measuring absorbance at OD_665_ for both the methanol extracted biofilm and the planktonic fraction of the culture.

### Bacterial adenylate cyclase two-hybrid assays

The bacterial adenylate cyclase two-hybrid assay (BACTH) [30,31] was employed to probe protein-protein interaction between various proteins. BTH101 (adenylate cyclase-deficient) *E. coli* strains transformed with appropriate plasmids were streaked onto Lysogeny Broth (LB) agar plates containing 100 µg ml^−1^ ampicillin and 50 µg ml^−1^ kanamycin and incubated at 30 °C for 24 h. Qualitative assays on MacConkey agar were performed as previously described [35], with several modifications as described [36].

### Microscopy

Light and fluorescence microscopy was performed with an EVOS M5000 fluorescence microscope (Life Technologies) equipped with a 10×, 40×, or 63× objective lens. Excitation and emission were as follows: EVOS light cube, Nrw 405 (AMEP4857: excitation 390/18 nm, emission 525/50 nm) for GFPuv; EVOS light cube, GFP (AMEP4651: excitation 470/22 nm, emission 525/50 nm) for UEA-fluorescein labelled HPS; EVOS Light Cube, DAPI (AMEP4650: excitation 357/44 nm, emission 447/60 nm) for immunofluorescence labelled PilA; and EVOS Light Cube, RFP (AMEP4652: excitation 531/40 nm, emission 593/40 nm) for cellular autofluorescence.

## Results

### Expression of ebsA is dependent on SigJ in developing hormogonia

**Fig. 1.**
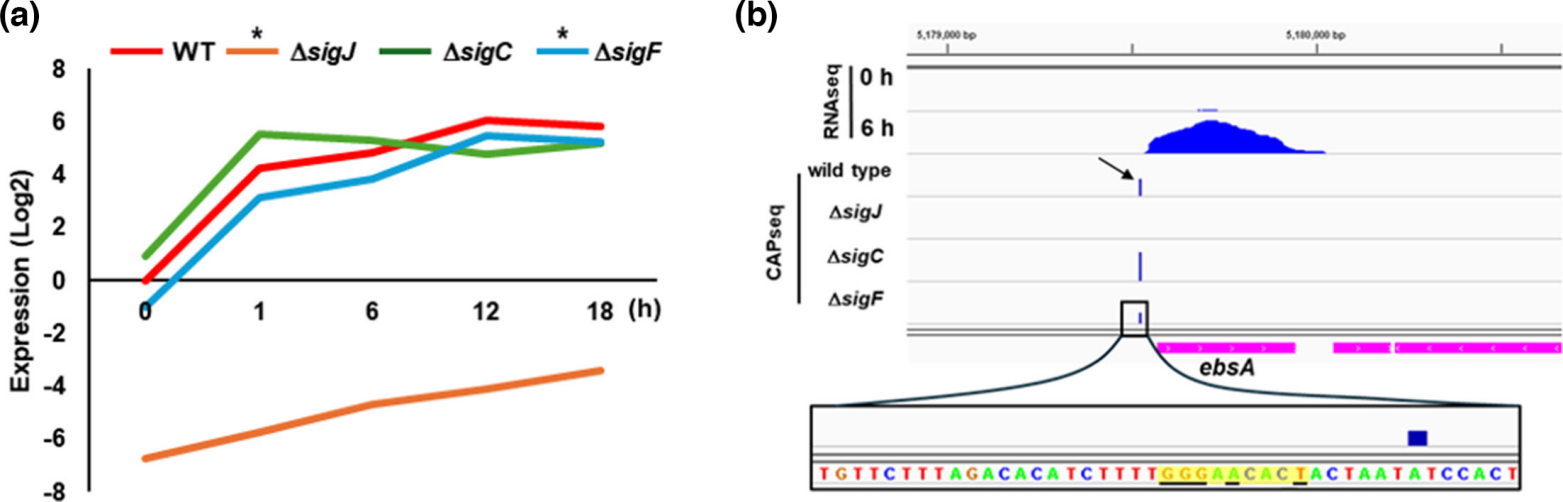
Expression of *ebsA* in developing hormogonia. (**a**) RNAseq based quantification of *ebsA* transcription in developing hormogonia (0–18 h post-induction) of the wild-type strain and hormogonium sigma factor mutants, derived from [38]. Expression=Experimental strain and time point/wild-type t=0. * indicates differential expression compared to the wild-type as determined by Bayesian Analysis of Time Series [42]. (**b**) Read coverage of the *ebsA* locus from RNAseq [38] and CAPseq [39] data sets (as indicated). Arrow indicates position of SigJ-dependent TSS. Inset below depicts nucleotide sequence upstream of the SigJ-dependent TSS with J-Box highlighted in yellow and absolutely conserved nucleotides of J-Box underlined.

Most heterocyst forming filamentous cyanobacteria only exhibit motility in differentiated filaments termed hormogonia, the development of which is regulated by the hybrid histidine kinase HrmX (formerly HrmK) [3,37] that in turn initiates a hierarchical sigma factor cascade [38]. To determine whether *ebsA* is specifically expressed in developing hormogonia of *Nostoc punctiforme* and which, if any, sigma factor is most directly responsible for *ebsA* regulation, previously published RNAseq [38] and Cappable-Seq (CAPseq) [39] data sets were analysed (Fig. 1a,b). Expression of *ebsA* was dramatically upregulated in developing hormogonia, reaching a maximum increase of 64-fold compared to vegetative filaments at 12 h post-hormogonium induction. Both *sigC-* and *sigF*-deletion strains displayed similar expression profiles for *ebsA* in developing hormogonia, although expression was slightly diminished in the *sigF* mutant. In contrast, expression of *ebsA* was dramatically reduced in vegetative filaments of the *sigJ*-deletion strain and although it did display a gradual increase in expression during hormogonium development, never approaches the levels seen even in vegetative filaments of the wild-strain. This is consistent with previous observations that SigJ-dependent promoters are still transcribed at low levels in vegetative filaments, and that deletion of *sigJ* or the upstream regulator *hrmX* results in even lower levels of expression for SigJ-dependent promoters in vegetative filaments [37,39]. These results indicate that expression of *ebsA* is most directly dependent on SigJ. CAPseq analysis detected a single transcriptional start site (TSS) in the promoter region for *ebsA* that was eliminated in the Δ*sigJ* strain. SigJ promoter recognition depends on the presence of a J-Box (GGGAATACT) [38,39] and the −10 region for this TSS contains a J-Box with only one nucleotide divergent from the consensus sequence. Collectively, these results indicate that *ebsA* is specifically expressed in developing hormogonia and that its transcription is directly dependent on SigJ. Previous work has indicated that there may be overlap in promoter recognition between SigJ- and SigF-dependent promoters [39], and this is consistent with the observation that transcription of *ebsA* in the RNAseq and CAPseq datasets are slightly reduced in the Δ*sigF* strain.

### EbsA is essential for motility, T4P extension and HPS secretion

**Fig. 2.**
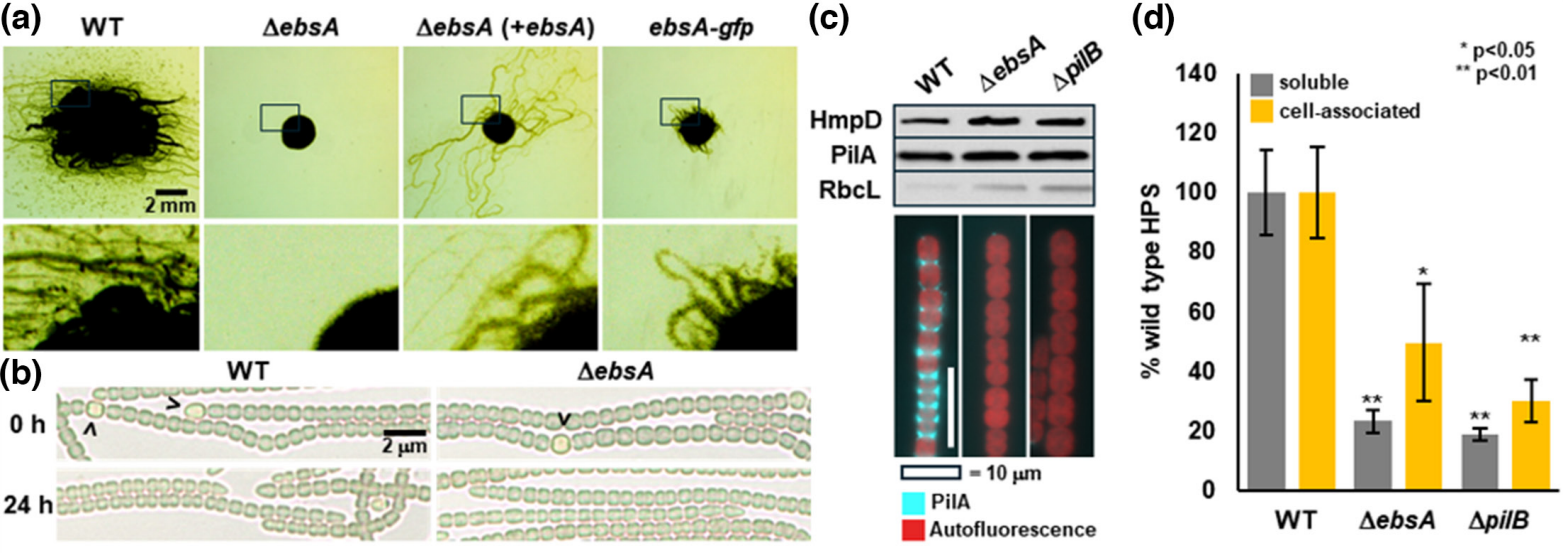
Characterization of the Δ*ebsA* strain. (**a**) Plate motility assays 5 d after transfer to medium promoting motility. Black boxes in row of upper images indicate magnified regions in lower row of images. (**b**) Light micrographs of vegetative filaments (0 h) and hormogonia (24 h post-induction) in the wild-type and Δ*ebsA* strain. Carets indicate heterocysts. Hormogonia are morphologically distinguished from vegetative filaments by the absence of heterocysts, smaller, more rod-shaped cells, and often tapered cells at the filament termini. (**c**) Immunoblot analysis of the hormogonium-specific proteins HmpD and PilA, and immunofluorescence analysis of surface-exposed PilA in the wild-type, Δ*ebsA*, and Δ*pilB* strains. RbcL serves as a protein loading control. (**d**) Quantification of cell-associated and soluble HPS by lectin-based analysis in the wild-type, Δ*ebsA*, and Δ*pilB* strains. *P*-values derived from students T-Test between the wild-type and each mutant strain.

To determine the precise role of *ebsA* in hormogonium motility, an in-frame deletion strain of *ebsA* was constructed in *N. punctiforme* and characterized. Deletion of *ebsA* completely abolished motility in plate and time-lapse assays (Figs 2a, S1, SMOV 1). The motility defect of the *ebsA* mutant could be restored by re-introduction of *ebsA*, expressed from the *petE* promoter on a shuttle vector, although the appearance of motility was delayed by ~2 days compared to the wild-type (Figs 2a, S1). This is consistent with numerous previous reports where complementation of motility-deficient mutants in this manner typically fails to completely restore motility to wild-type levels and is likely due to gene dosage effects (see [38] for discussion). When the chromosomal allele of *ebsA* was replaced with an allele producing EbsA with GFP fused to the C-terminus, motility was severely reduced, although detectable compared to the Δ*ebsA* strain, indicating that the fusion protein retains at least partial function (Figs 2a, S1). Despite the absence of motility, the Δ*ebsA* strain developed hormogonium filaments that retained the morphology of wild-type hormogonia (Fig. 2b), indicating that *ebsA* is not essential for early stages of hormogonium development.

To further determine the effect of *ebsA* on the gene regulatory network controlling hormogonium development and T4P activity, immunoblot analysis was performed to assess the expression of the hormogonium specific proteins HmpD and PilA, along with immunofluorescence analysis of PilA to assess surface piliation. Because the current evidence indicates that EbsA is part of a complex with PilB and is essential for T4P function, a previously generated Δ*pilB* strain [11] was included in the analysis for comparison. Deletion of *ebsA* did not affect the accumulation of HmpD or PilA in developing hormogonia but eliminated the presence of PilA on the cell surface, similar to the phenotype observed for the Δ*pilB* strain (Figs 2c, S2). Both the complemented Δ*ebsA* strain, and the *ebsA-gfp* strain exhibited detectable surface PilA (Fig. S2), although the levels of surface PilA appeared severely reduced in the *ebsA-gfp* strain, consistent with the hypothesis that the presence of the GFP tag on EbsA disrupts, but does not abolish EbsA function, and with the observation that motility is severely reduced in the *ebsA-gfp* strain. These results are consistent with those reported for unicellular cyanobacteria, indicating that *ebsA* is essential for T4P extension. In addition to T4P activity, hormogonium motility requires the deposition of hormogonium polysaccharide (HPS), and previous studies have demonstrated that mutation of other genes required for T4P extension, such as *pilB* and *hfq*, abolish HPS secretion [11,16]. To determine if HPS secretion is disrupted in the Δ*ebsA* strain, lectin blotting and fluorescent-lectin staining were employed to quantify the soluble and cell-associated fractions of HPS, respectively. Deletion of *ebsA* dramatically reduced the accumulation of HPS in a manner similar to the Δ*pilB* strain (Fig. 2d). In fluorescent lectin staining, only weak fluorescence was detected, associated with detached heterocysts (Fig. S3), consistent with previous observations for a Δ*pilB* strain [11]. Overall, the phenotype of the Δ*ebsA* strain is essentially indistinguishable from that of a Δ*pilB* strain, consistent with the findings in unicellular cyanobacteria [23], and support a model where EbsA is essential, along with PilB, for both pilus extension and HPS secretion.

### Localization of EbsA and interaction between EbsA and other T4P proteins

**Fig. 3.**
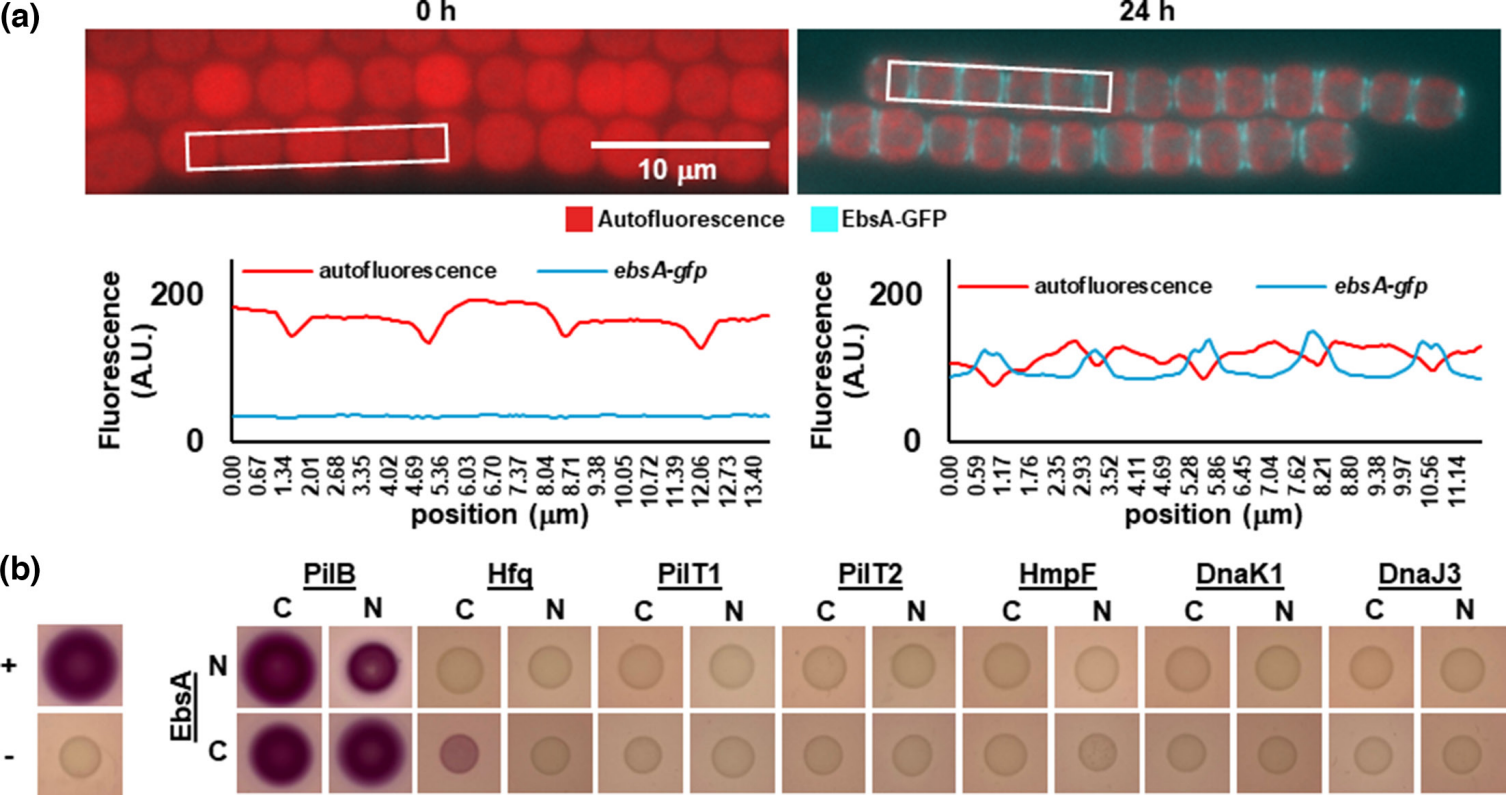
Localization of EbsA-GFP and protein-protein interaction between EbsA and T4P components. (**a**) Fluorescence micrographs and quantification of fluorescence of vegetative (0 h) and hormogonium (24 h post-induction) filaments of the *ebsA-gfp* strain. White boxes indicate regions where fluorescence was quantified. (**b**) BACTH assays testing for interaction between EbsA with N- or C-terminal fusions to the T25 fragment of adenylate cyclase, and various T4P proteins (as indicated) with N- or C-terminal fusions to the T18 fragment of adenylate cyclase.

In *N. punctiforme*, the T4P systems are arrayed in rings at each end of the cell, adjacent to the septum. Fluorescent protein fusions have demonstrated that most T4P components, including PilB and Hfq, are statically localized to these rings at both cell poles [11,16]. To determine if EbsA exhibits a similar localization pattern, fluorescence microscopy was employed to visualize the localization of EbsA-GFP (Fig. 3a). In vegetative filaments, GFP-derived fluorescence was undetectable, consistent with the expression data presented above, indicating that *ebsA* is transcribed specifically in hormogonia. In hormogonia, EbsA-GFP was clearly visible and displayed static, bi-polar localization like that reported previously for PilB and Hfq. Immunoblot analysis with α-GFP antibodies confirmed the presence of a full length EbsA-GFP fusion protein (Fig. S4) and corroborated that EbsA-GFP levels are extremely low in vegetative filaments. This finding supports previous studies indicating that EbsA is part of a protein complex along with PilB and Hfq and implies that this complex is statically localized to both cell poles in *N. punctiforme*.

While the previous work on EbsA demonstrated that it is part of a protein complex that includes both Hfq and PilB this previous study [23] did not resolve which proteins in the complex interact directly versus indirectly. Moreover, this study did not test for interaction with other proteins associated with the T4P system, in particular proteins such as HmpF or the pilus-associated chaperone system protein DnaK1. Because the association of these proteins with the T4P motor is dynamic in nature, and these proteins dissociate from the T4P system in response to chemical treatments that alter membrane polarity [16,21], it is unlikely that they would be remain associated with the T4P systems following cell lysis and therefore may not be detected using coimmunoprecipitation. To resolve the protein-protein interaction network between EbsA, Hfq, and PilB, and to determine whether EbsA interacts with any other components of the T4P system in *N. punctiforme*, the bacterial adenylate cyclase two-hybrid (BACTH) system [30] was employed.

BACTH assays clearly imply that EbsA interacts directly with PilB, as all possible combinations of N- and C-terminal fusions of the adenylate cyclase domains to these proteins produced a colour change on MacConkey medium comparable to the positive control (Fig. 3b). In contrast, only a very weak colour change was observed for a single pairing of N- and C-terminal fusions for EbsA and Hfq (Fig. 3b). Thus, it is possible that EbsA and Hfq may directly interact, but alternative experimental approaches would be required to confirm this. In contrast, no interaction was detected between EbsA and either PilT1, PilT2, HmpF, DnaK1, or DnaJ3 (Fig. 3b). These findings further resolve the protein-protein interaction network of these T4P system components and imply that EbsA does not serve as a target for transient association of HmpF or DnaK1, like Hfq does [16,21].

### *N. punctiforme ebs*A is essential for biofilm formation

**Fig. 4.**
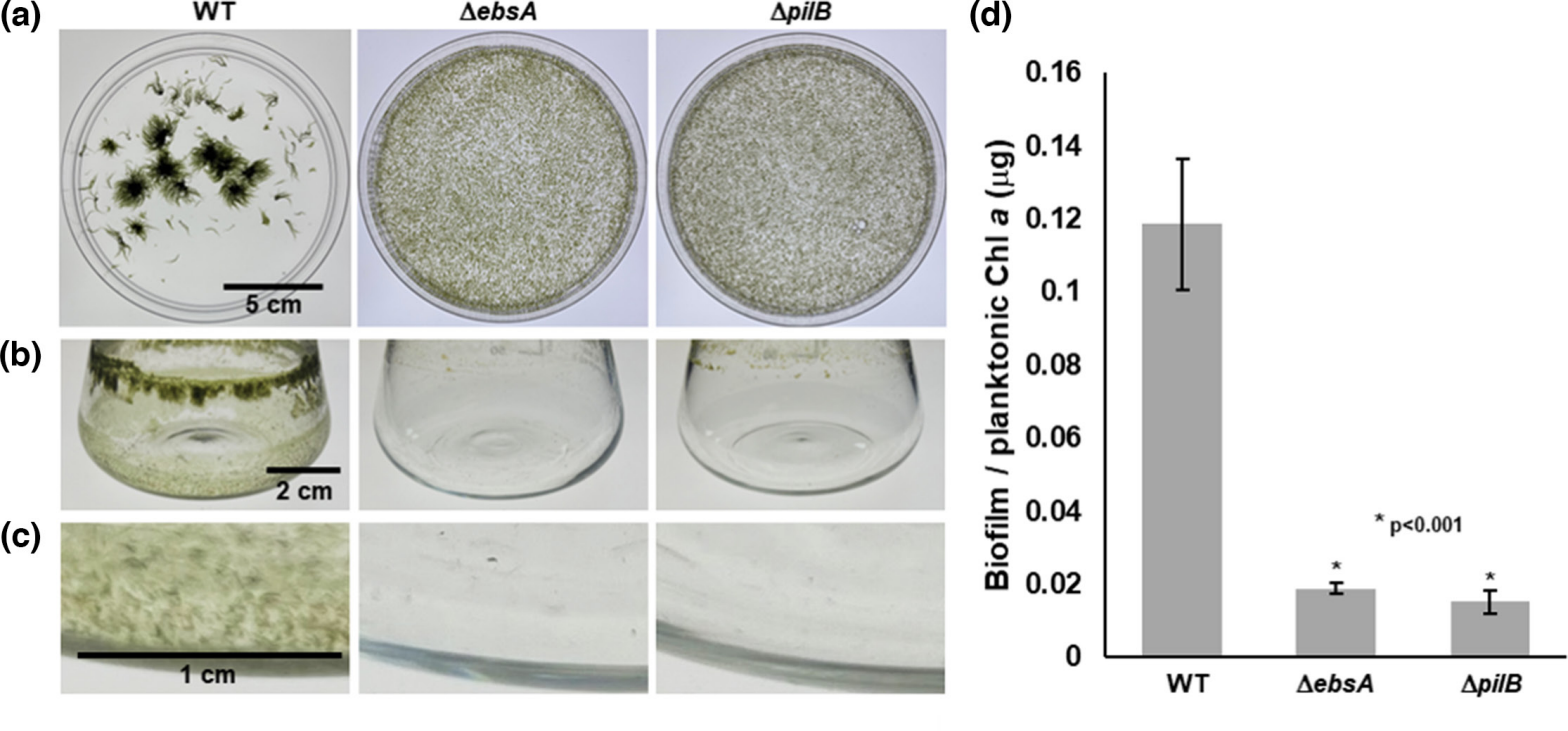
Biofilm formation in the wild-type, Δ*ebsA*, and Δ*pilB* strains. (**a**) Images of liquid cultures not dispersed by pipetting, transferred to empty petri plates to facilitate better imaging, for strains as indicated. (**b**) Images of culture vessels from cultures dispersed by routine pipetting, following removal of liquid culture and washing of the vessel. (**c**) Close-up view of bottom side of culture vessels. Strain labels the same for a–c. (**d**) Quantification of biofilms based on extraction of Chl *a* from cells adhering to culture vessel normalized against Chl *a* from the planktonic fraction. *P*-values derived from students T-Test between the wild-type and each mutant strain.

In liquid culture, wild-type *N. punctiforme* forms colonial aggregates [5]. We have also observed the formation of biofilms in culture vessels, especially when the aggregates are disrupted by routine, vigorous pipetting, but we have not systematically investigated this or reported these findings elsewhere. Generally, we have observed that mutations disrupting motility lead to the loss of colonial aggregate formation, as has been reported for a Δ*hmpD* strain [5]. In contrast, in *S. elongatus*, mutation of *ebsA* or *pilB* led to an increase in biofilm formation [23]. Thus, we performed experiments to systematically investigate the biofilm forming capacity of wild-type *N. punctiforme* and that of the Δ*ebsA* and Δ*pilB* strains. Under standard culture conditions, the wild-type strain forms colonial aggregates (Fig. 4a). However, upon routine disruption of these aggregates by pipetting, biofilms form on the bottom and sides of the culture vessel (Fig. 4b–d). No pellicles were observed on the surface of the liquid medium. Biofilm formation was most pronounced on the side of the flask near the liquid-air interface but was also present well below the surface of the liquid on the bottom and sides of the flask. In contrast, both the Δ*ebsA* and Δ*pilB* strains produce very dispersed cultures and show no obvious signs of biofilm formation, with only small amounts of cell material accumulating at the liquid-air interface and virtually no accumulation below the surface of the liquid medium (Fig. 4a–d). These results indicate that unlike in *S. elongatus*, *ebsA* and *pilB* are essential for biofilm formation in *N. punctiforme*.

## Discussion

The data presented here supports the hypothesis that EbsA is a core component of the pilus extension motor, along with Hfq and PilB. Like Hfq and PilB [11,16], EbsA-GFP displays static, bipolar localization, and two-hybrid analysis indicated a direct interaction between EbsA and PilB. It should be noted that given the low functionality of the EbsA-GFP fusion protein, we cannot completely rule out the possibility that the presence of the GFP tag altered the normal localization of EbsA. However, given the similarity of EbsA localization to that of PilB and Hfq, it seems most likely that these form stable complexes that reside at both poles. Given that the two-hybrid analysis failed to detect any interactions between EbsA and either PilT1 or PilT2, it seems unlikely that EbsA is involved in pilus retraction. Likewise, the failure to detect interaction between EbsA and HmpF, DnaK1, or DnaJ3 indicate that EbsA is not a target for interaction with HmpF to activate the T4P systems, as previously observed for Hfq and HmpF [16], nor is EbsA directly involved in the DnaK1/J3 chaperone system.

In contrast to the role of *ebsA* in T4P activity and motility, which appears to be conserved in motile cyanobacteria, its role in biofilm formation is substantially different in those species where it has been investigated thus far. In S. elongatus, *ebsA* suppresses biofilm formation, while in *Synechocystis ebsA* has no apparent effect on biofilm formation [23]. In contrast, *ebsA* is essential for both motility and biofilm formation in *N. punctiforme*. This is consistent with observations in other filamentous cyanobacteria that aggregation [4,7] and biofilm formation [25] require motility. Given that deletion of *ebsA* in *N. punctiforme* results in the loss of surface piliation and HPS secretion, it would appear that one or both of these are critical components of the biofilms formed by *N. punctiforme* and by extension, possibly other filamentous cyanobacteria. This is consistent with the observation that both T4P and exopolysaccharide are critical components of biofilms in a broad range of bacteria [40]. These findings could indicate that very distinct modalities exist for biofilm formation in different cyanobacteria. Alternatively, it is possible that the effect of *ebsA* on biofilm formation observed in *S. elongatus* could be due to dysregulation of the underlying genetic system controlling this process because of mutations acquired in the lab. Unlike the laboratory type strain of *S. elongatus* which is non-motile and does not form biofilms, a recently characterized wild isolate of the same organism displays both motility and biofilm formation [24]. Given these findings, mutation of *ebsA* in this wild isolate could provide more insight on the relationship between *ebsA* and biofilm formation in cyanobacteria.

The fact that both colonial aggregation and biofilm formation in *N. punctiforme* require the same genetic factors indicates that the colonial aggregates observed for *N. punctiforme* and many other filamentous cyanobacteria should be regarded as non-surface attached biofilms. Recent work has begun to recognize that aggregation in liquid phase involves the same physiological processes as surface attached biofilm formation, and therefore aggregation in liquid can be considered a biofilm where the bacterium themselves form the surface [41].

## Abbreviations

AA/4: Allan and Arnon medium diluted 4-fold
Chl a: chlorophyll a
GFP: green fluorescent protein
HPS: hormogonium polysaccharide
HRP: horse radish peroxidase
T4P: type IV pilus
UEA: Ulex Europus Agglutinin I
